# Correlation Between Post-operative Sense of Coherence and Family Function in Patients With Type A Aortic Dissection

**DOI:** 10.3389/fsurg.2022.857219

**Published:** 2022-04-14

**Authors:** Xiaomin Zhang, Juan Chen, Yaning Zang

**Affiliations:** ^1^Department of Cardiothoracic Surgery, Nanjing First Hospital, Nanjing Medical University, Nanjing, China; ^2^The Third School of Clinical Medicine, Nanjing Medical University, Nanjing, China

**Keywords:** type A aortic dissection, sense of coherence, family function, correlation, research

## Abstract

**Objective:**

To analyze the relationship between post-operative sense of coherence and family function in patients with type A aortic dissection (AD).

**Methods:**

Ninety patients with AD treated from January 2019 to December 2020 were selected as the research subjects. All patients received surgical treatments. Two weeks after the operation, the Sense of Coherence Scale (SOC-13) and Family APGAR index scale (APGAR) were used to evaluate the patients' sense of coherence and family function. Baseline data of all patients were collected, the SOC-13 scores of patients with type A AD with different demographic characteristics were compared, and the relationship between family function and patients' sense of coherence was analyzed.

**Results:**

The ninety patients with type A AD had a low level of psychological consistency, and the average SOC-13 score was 49.84 ± 3.89 points. The SOC-13 score of patients with type A AD with family monthly incomes <5,000 yuan and moderate and severe family dysfunction was lower than that of patients with family monthly incomes ≥5,000 yuan and good family function. The difference was statistically significant (*P* < 0.05). There was no statistically significant difference in the SOC-13 scores of patients with type A AD with different demographic characteristics (*P* > 0.05). The results of multiple linear regression analysis showed that family monthly income <5,000 yuan and moderate and severe family dysfunction might be general influencing factors of sense of coherence among patients with type A AD (*P* < 0.05). Y = 43.333 + 6.667X_1_ + 16.730X_2_ was obtained.

**Conclusion:**

The post-operative sense of coherence of patients with type A AD may be affected by family function.

## Introduction

As the preferred treatment for patients with type A aortic dissection (AD), surgical treatment aims to prevent aortic rupture and cardiac tamponade, improve patient hemodynamics and reduce mortality in patients with type A AD (1). Due to the serious condition of type A AD, the patient may have persistent pain and need lifelong antihypertensive therapy after the operation (2, 3). Psychological congruence is the individual's perception of life as a whole, and it can reflect the individual's internal psychological tendency (4). Research indicates that the level of psychological congruence can influence the self-management ability of patients and improve their quality of life (5). Studies in China and internationally have shown that (6–9) among post-operative AD patients, a psychological status oriented toward improving quality of life, especially for long-term (6 months or more) quality of life, is positively related to a good psychological state; patients with a better psychological status have a better understanding of stressors and a better physical status, actively engage in health-related behavior, have better self-management ability, and thus have a better quality of life. Research by Fanghui Lou (10) and Zhao Rong (11) showed that psychological intervention for patients after AD can correct negative emotions, improve psychological status, promote patients' sense of psychological consistency and improve their quality of life. Therefore, it is important to clarify the status quo of psychological congruence in patients with type A AD and to determine the related factors for improving their quality of life. Family function mainly refers to the family's ability to satisfy various psychological and physiological development needs of family members (12). Studies have shown that good family functioning contributes to patients' self-management ability (13). Combined with the influence of the abovementioned psychological congruence and family function on the self-management ability of patients with type A AD, it is inferred that there may be some relationship between psychological congruence and family function. In view of this, this study focuses on a correlation analysis of type A AD with post-operative psychological coherence and family function. The report is as follows.

## Materials and Methods

### General Data

Ninety patients with AD admitted from January 2019 to December 2020 were selected as subjects for this study, which was approved by the hospital ethics committee. All patients and their families were informed about the study and voluntarily signed the informed consent form. Among the 90 patients, 64 were male, and 26 were female. The average age was 40–75 years (57.82 ± 3.58 years). Regarding education level, 28 patients were educated at the junior high school level or below, 39 patients were educated at the senior high school level or technical secondary school, and 23 patients were educated at the junior college level or above.

### Inclusion and Exclusion Criteria

#### The Inclusion Criteria Were as Follows

① Patients had AD that met the relevant standards in internal medicine (9th Edition) (14) and was diagnosed as Stanford type A AD by echocardiography and enhanced vascular CT; ② patients had surgical treatments, including the Bentall operation, Wheat operation or ascending aorta replacement, that were performed for the first time; and ③ patients had good communication and comprehension skills and were able to complete the questionnaire independently.

#### The Exclusion Criteria Were as Follows

① Patients had complicated AD with serious complications, such as respiratory failure, cardiac respiratory arrest, renal failure, cerebral infarction, cerebral hemorrhage, coma, shock, or heart failure; ② patients had AD combined with malignant tumor diseases such as liver cancer or lung cancer; ③ patients had mental illness or cognitive impairment and were unable to cooperate with the investigation; or ④ patients withdrew or discontinued their participation halfway through the study.

### Methods

#### Assessment of Psychological Consistency

Psychological consistency was evaluated 2 weeks after the operation, and the Sense of Coherence scale (SOC-13) (15) was used to evaluate the psychological consistency of patients. The scale includes 3 dimensions, including sense of control (items 3, 4, 5 and 10, with a total score of 4–28 points), sense of understanding (items 2, 6, 7, 8, and 12, with a total score of 5–35 points), and sense of meaning (items 1, 9, 11, and 13, with a total score of 4–28 points). Each item is scored from 1 to 7 points according to 7 levels, and the total score range is 13–91 points. The higher the score is, the higher the level of psychological consistency of patients. According to the scores, the level of patients' sense of psychological consistency was classified, with scores of 13–63 being classified as a low level, scores of 64–79 being classified as a medium level, and scores of 80–91 being classified as a high level. Patients selected each item in the questionnaire according to their own situations, and the score of each item ranged from 1 to 7 points. The researcher summed the scores for each item according to the patient's responses, and the final total score was the patient's sense of psychological consistency score (Table 1).

**Table 1:** Sense of coherence-13, SOC-13.

**Here is a life orientation scale with seven different answers to each question, ranging from less to more severe. Please tick “√” according to your own feelings**.		
**Item**	**Option**	**Score**
1. Do you often feel like you don't care what's going on around you?	① never ② less ③ very little ④ hard to say ⑤ sometimes ⑥ more ⑦ often	
2. How often in the past have you been surprised by someone you thought you knew well?	① never ② less ③ very little ④ hard to say ⑤ sometimes ⑥ more ⑦ often	
3. How often has someone you count on let you down?	① never ② less ③ very little ④ hard to say ⑤ sometimes ⑥ more ⑦ often	
4. Do you often feel that you are being treated unfairly?	① never ② less ③ very little ④ hard to say ⑤ sometimes ⑥ more ⑦ often	
5. Do you often feel like you're in strange situations and don't know what to do?	① often ② sometimes ③ at individual times ④ I don't know ⑤ less ⑥ seldom ⑦ hardly or never	
6. Do you often have very complicated, mixed feelings and thoughts?	① very often ② often ③ sometimes ④ I don't know ⑤ less ⑥ seldom ⑦ hardly or never	
7. Do you often feel emotions you don't want to feel?	① very often ② often ③ sometimes ④ I don't know ⑤ less ⑥ seldom ⑦ hardly or never	
8. Many people, even very talented people, sometimes feel like failures under certain circumstances. Have you experienced this often in the past?	① never ② less ③ very few ④ hard to say ⑤ sometimes ⑥ more ⑦ often	
9. How often do you think that doing these things every day doesn't mean anything?	① very often ② often ③ sometimes ④ I don't know ⑤ less ⑥ seldom ⑦ hardly or never	
10. Do you often feel out of control?	① very often ② often ③ sometimes ④ I don't know ⑤ less ⑥ seldom ⑦ hardly or never	
11. Your life so far:	① no goals at all ② no goals ③ not very purposeful ④ unclear? have a life goal ⑤ clear life purpose ⑥ very clear life purpose	
12. When confronted with a problem or issue, you often find yourself—	① underestimating or overestimating its importance ② finding the matter hard to assess ③ addressing the matter slightly inaccurately ④ finding the matter hard to grasp ⑤ assessing the matter somewhat accurately ⑥ assessing the matter accurately ⑦ assessing the matter very accurately	
13. Doing the things you do every day—	① brings a great deal of happiness and satisfaction ② makes me happier and more satisfied ③ makes me a little happy ④ I don't know ⑤ makes me a little unhappy ⑥ makes me very unhappy? is a source of pain and trouble	
Total points		

#### Family Function Assessment

Two weeks after the operation, the Family APGAR index (APGAR) (16) was used to assess the patient's family function. The scale consists of five dimensions: cooperation, emotion, adaptability, intimacy and growth. Each item is scored as 2, 1, or 0, which correspond to frequently, sometimes or rarely, respectively. The total score range is 0–10. The higher the score is, the better the family function. A total score ≤3 points indicates severe impairment of family function, 4–6 points indicates moderate impairment of family function, and ≥7 points indicates good family function. Patients rated each item of the scale according to their own situations, and the score of each item ranged from 0 to 2 points. The researcher summed the scores of the 5 items according to the patient's responses, and the final total score was the patient's family function score (Table 2).

**Table 2:** Family care index questionnaire (APGAR).

**Item**	**Often (2 points)**	**Sometimes (1 point)**	**Hardly ever (0 point)**
When I have problems, I can get satisfactory help from my family.	□	□	□
I am very satisfied with the way my family discusses various things and shares problems with me.	□	□	□
When I wish to pursue new activities or developments, my family is receptive and supportive.	□	□	□
I am satisfied with the way my family shows concern and love for my emotions (joy, anger, sorrow, joy).	□	□	□
I'm happy with the way my family spends time with me.	□	□	□

#### Baseline Data Collection Design

A baseline data questionnaire was administered to collect baseline data of all patients, including age (<60 years old, ≥60 years old), sex (male and female), education level (junior middle school and below, senior high school or technical secondary school, college and above), marital status (married, unmarried/divorced/widowed), working status (employed, retired), care (family members, others, no care), and family monthly income (<5,000 yuan, ≥5,000 yuan).

#### Quality Control

Before the study questionnaire was distributed, the purpose and reporting method of the survey was explained to the patients. The questionnaire was evenly distributed to the patients by two staff members to ensure that the patients completed the questionnaire independently within 30 min. After completion, the staff collected the questionnaire, checked whether the questionnaire was valid and eliminated invalid questionnaires (either all items were the same answer or there was a regular pattern of answers), included the valid questionnaires and entered the data. A total of 90 questionnaires were distributed, and 90 were recovered, for a recovery rate of 100%.

#### Statistical Methods

The data were processed by SPSS 22.0 software. The measurement data are expressed with respect to the normal distribution proposed by the Shapiro–Wilk normal distribution test. The independent sample *t*-test was used for intergroup comparisons, the one-way variance test was used for multigroup data, and the SNK test was used for pairwise comparisons. Multiple linear regression analysis was used to test the effect of family function on psychological consistency in patients with type A AD; *P* < 0.05 was considered statistically significant.

## Results

### Post-operative SOC-13 Score of Patients With Type A AD

The ninety patients with type A AD had a low level of psychological consistency. The average SOC-13 score was 49.84 ± 3.89.

### Comparison of SOC-13 Scores of Patients With Type A AD With Different Demographic Characteristics

The SOC-13 score of patients with type A AD with family monthly incomes <5,000 yuan and moderate and severe family dysfunction was lower than that of patients with family monthly incomes ≥5,000 yuan and good family function (*P* < 0.05). There was no significant difference in SOC-13 scores among patients with type A AD with different demographic characteristics (*P* > 0.05) (Table 3).

**Table 3:** Comparison of the SOC-13 scores of patients with type A AD with different demographic characteristics.

**Characteristics**		***n***	**SOC-13 score (points)**	**Statistical value**	***P***
Age	<60 years old	68	49.77 ± 3.46	*t* = 0.322	0.748
	≥60 years old	22	50.06 ± 3.81		
Gender	Male	64	49.89 ± 2.98	*t* = 0.281	0.779
	Female	26	49.72 ± 3.16		
Education level	Junior high school and below	28	49.22 ± 2.95	*F* = 0.641	0.529
	High school or technical secondary school	39	50.14 ± 3.56		
	College degree or above	23	50.09 ± 3.99		
Marital status	married	69	49.52 ± 3.65	*t* = 1.473	0.144
	Unmarried/divorced/widowed	21	50.89 ± 3.58		
Working status	On the job	68	50.11 ± 3.91	*t* = 1.171	0.245
	Retired	22	49.01 ± 3.63		
Care situation	Family members	62	50.08 ± 3.09	*F* = 0.831	0.439
	Someone else	22	49.58 ± 4.17		
	Unattended	6	48.31 ± 3.25		
Monthly household income	<5,000 yuan	9	32.58 ± 2.70	*t* = 11.383	<0.001
	≥5,000 yuan	81	51.76 ± 4.96		
Family function	Good family function	63	60.21 ± 5.78	*F* = 449.560	<0.001
	Moderate impairment of family function	19	25.41 ± 2.09^a^		
	Severe family dysfunction	8	26.20 ± 2.25^a^		

*Compared with family function*,

a *the difference is statistically significant*.

### Linear Regression Analysis of the Influence of Various Factors on the Sense of Psychological Consistency of Patients With Type A AD

Taking the psychological consistency (SOC-13 score) of patients with type A AD as the dependent variable and the variable with a statistically significant difference of 2.2 as the independent variable, the equation was obtained by multiple linear regression analysis: *y* = 43.333 + 6.667x1 + 16.730x2, in which the F value of the regression model was 70.064, R2 was 0.617, and the adjusted R2 was 0.608. The results showed that family monthly income <5,000 yuan and moderate and severe family dysfunction may be influencing factors of general psychological consistency in patients with type A AD (*t* = 2.301, 8.822, all *P* < 0.05) (Table 4).

**Table 4:** Linear regression analysis of the influence of various factors on the sense of psychological consistency of patients with type A AD.

**Factor**	**B value**	**βvalue**	**95% CI of B value**	***t***	***P***
Constant	43.333	2.365	38.632–48.035	18.321	0.001
Monthly household income (X_1_)	6.667	2.897	0.909–12.424	2.301	0.024
Family function (X_2_)	16.730	1.896	12.961–20.499	8.822	0.001

## Discussion

Affected by pain associated with the disease, surgical stress and disease management, patients with type A AD have poor psychological adaptability, low confidence in rehabilitation, an inability to independently adjust their psychological emotions and coping styles, a low level of psychological consistency, poor self-management ability and poor post-operative rehabilitation effects (17). The results of this study showed that the level of psychological consistency was low in the 90 patients with type A AD, suggesting that post-operative psychological consistency among type A AD patients is generally low. Therefore, it is important to analyze the relevant factors affecting the post-operative psychological consistency of patients with type A AD and to take corresponding measures to improve the rehabilitation effects of patients with type A AD.

A sense of psychological consistency mainly refers to the individual's tendency to maintain self-confidence when faced with stress and pressure, which is mainly reflected in his or her understanding of stressors and pressure and perception of his or her own coping ability (18). A sense of psychological consistency includes a sense of control, a sense of understanding and a sense of meaning, in which the sense of control refers to the individual's self-confidence in dealing with problems, the sense of understanding refers to the individual's recognition of the logic of events from the internal and external environment, and the sense of meaning refers to the individual's subjective judgment and understanding of events. The three components work together to exert a positive psychological effect so that patients can correctly perceive stress and stressful events and respond positively (19). Family function is a comprehensive evaluation index of the family. On the one hand, it indicates whether the basic material needs of family members are being met, and on the other hand, it reflects the emotional communication and family cohesion between family members. It mainly refers to the overall self-perceived ability of the family to deal with pressure and suffering (20). One study noted that patients with good family function could mobilize more family resources and that family members could provide additional care, which is conducive to enhancing patients' confidence in coping with diseases and helping them change their coping styles (21). Therefore, it is preliminarily speculated that family functioning may affect patients' sense of psychological consistency. The results of multiple linear regression analysis show that family function can affect the post-operative psychological consistency of patients with type A AD. The reason is that patients with close emotional ties among family members can better express their post-operative psychological emotions. With the help of family members, patients' psychological adaptability is improved, and their acceptance of the disease is improved, which is conducive to helping patients establish confidence in rehabilitation (22). On the other hand, good family functioning is conducive to improving patients' ability to manage disease. Mutual support and the sharing of resources among family members can facilitate patients' disease management to improve patients' self-management ability, and the sharing of responsibilities can actively mobilize patients' subjective initiative and improve their sense of psychological consistency (23). Good family function can create a comfortable rehabilitation environment for patients, offer patients physiological care and psychological comfort, encourage patients' physiological and psychological rehabilitation, help patients endure pressure and stressors, reduce psychological burden, and improve patients' sense of psychological consistency (24, 25). In addition, this study also found that family monthly income can affect patients' sense of psychological consistency, which may be related to the fact that treatment and rehabilitation costs can increase patients' psychological burden on the family.

The relevant research shows that (26, 27) researchers have mainly conducted family functional intervention through cognitive behavioral therapy, family psychological education, family membership and so on. Cognitive behavioral interventions are mainly performed through health development and the distribution of manuals, health lectures, personalized communication, etc. Family psychological education is mainly based on multidisciplinary cooperation and family members' participatory care, providing rehabilitation knowledge to patients. Disease-related knowledge should be disseminated to major family caregivers, medical caregivers, nursing caregivers and family members so they can jointly formulate personalized care plans. At the same time, disease care and other related help should be provided via WeChat, telephone, the internet and other ways. Intervention with family members benefits both patients and all family members, and meetings should be held to address disease-related impacts and problems. Such impacts include the impact of the illness on family members' lives, psychological pressure and economic burden, role changes and the ability to deal with emergencies. Through different interventions, the cognition of patients and family members can be improved; the relationship, psychological state and coping ability of family members can be improved; the intimacy of family members can be increased; and family function can be improved.

## Conclusion

In conclusion, the post-operative psychological consistency of patients with type A AD is related to family function. There are various clinical interventions for family function. It is suggested that personalized interventions be selected according to the particularities of type A AD patients to meet patients' needs for family support, mobilize family function, and improve patients' sense of psychological consistency.

## Data Availability Statement

The raw data supporting the conclusions of this article will be made available by the authors, without undue reservation.

## Ethics Statement

Ethical review and approval was not required for the study on human participants in accordance with the local legislation and institutional requirements. The patients/participants provided their written informed consent to participate in this study.

## Author Contributions

XZ and JC conceived of and designed the study. XZ and YZ collected and analyzed the data. XZ drafted the manuscript. All authors have read and approved the final manuscript.

## Conflict of Interest

The authors declare that the research was conducted in the absence of any commercial or financial relationships that could be construed as a potential conflict of interest.

## Publisher's Note

All claims expressed in this article are solely those of the authors and do not necessarily represent those of their affiliated organizations, or those of the publisher, the editors and the reviewers. Any product that may be evaluated in this article, or claim that may be made by its manufacturer, is not guaranteed or endorsed by the publisher.
